# Draft genome sequences of biofuel production microalgae *Auxenochlorella protothecoides* and *Chlorella sorokiniana*

**DOI:** 10.1128/MRA.00440-23

**Published:** 2023-11-30

**Authors:** P. S. Shwed, S. Bernatchez, G. Leveque

**Affiliations:** 1 Environmental and Radiation Health Sciences Directorate, Healthy Environments and Consumer Safety Branch, Ottawa, Canada; 2 Biotechnology Section, New Substances Control and Assessment Bureau, Health Canada, Ottawa, Canada; 3 Canadian Centre for Computational Genomics (C3G), McGill University, Montréal, Canada; University of Maryland School of Medicine, Baltimore, Maryland, USA

**Keywords:** microalgae, protists, shotgun, DNA sequencing

## Abstract

Green microalgae are used in biofuel production. To contribute to the knowledge of the Chlorellaceae family, the *de novo* assembled draft genome sequences of *Auxenochlorella protothecoides* Kruger and *Chlorella sorokiniana* Shihira and Krauss are presented.

## ANNOUNCEMENT

Microalgal strains of the family Chlorellaceae are photosynthetic and can also metabolize both inorganic and organic carbon sources. They are also of interest for various biotechnology applications including food production (1 – 3). The draft genome sequences of two members of the Chlorellaceae family, presented here, have been collected as part of research to support the regulation of products of biotechnology in Canada.

*Auxenochlorella protothecoides* ATCC 30407, formerly *Chlorella protothecoides* (4), and *C. sorokiniana* ATCC 22521 were obtained from the ATCC. *A. protothecoides* is a sap wound isolate from a poplar tree and was grown on ATCC medium 847 agar. *C. sorokiniana* was isolated from warm surface waters near the coast of Texas (USA) and grown on ATCC medium 5 agar. Both strains were cultivated at 25°C with a 14-hour light/10-hour dark cycle, using full-spectrum LED lighting. Genomic DNA from both organisms was extracted using the DNeasy PowerLyzer PowerSoil Kit (Qiagen, USA). DNA libraries were prepared (NEB Next Ultra II) and sequenced with the HiSeq 4000 platform (Illumina) and the paired-end 100-bp protocol. Reads were trimmed for quality and adaptor sequence using fastp-v.0.19.7 (5). *De novo* assemblies were carried out with SPAdes-3.15.4 using the default parameters as well as the “ --cov-cutoff auto” option (6). BBMap v38.90 (https://sourceforge.net/projects/bbmap/) was used to calculate the assembly scaffolds coverage.

The assembly for *A. protothecoides* had 4,533 contigs with an N50 value of 87,325 bp, and the largest contig was 359,649 bp. The average read coverage was 1,193-fold. The assembly for *C. sorokiniana* contained 7,395 contigs with an N50 value of 202,422 bp and the largest contig at 677,079 bp. The average read coverage was 520-fold.

Both assemblies were judged as nearly complete by BUSCO v3.1.0 analysis using default settings and the “chlorophyta_odb10” data set that contained 2,168 benchmarking universal single-copy orthologs (7). *A. protothecoides* was analyzed as 95.7% complete, 1.3% fragmented, and 3% missing, whereas *C. sorokiniana* was 92.6% complete, 2.4% fragmented, and 5.0% missing. The GC content of *A. protothecoides* ATCC 30407 and *C. sorokiniana* ATCC 22521 were 63.8% and 63.9%, respectively.

Genome k-mer frequency histograms of trimmed Illumina R1 fastq files were derived by Jellyfish 2.2.6 (8) with the settings: -C -m 21 s 400000000. Genome characteristics were estimated from k-mer frequency histograms with Genomescope 2.0, using default parameters (9). *A. protothecoides* ATCC 30407 and *C. sorokiniana* ATCC 22521 haploid genome sizes were estimated as 56.9 Mb and 21.4 Mb, respectively.

These two draft genomes for the family Chlorellaceae will contribute as resources for genome comparisons and for predictive metabolic analyses.

## Data Availability

The draft genome sequences of *A. protothecoides* Kruger and of *C. sorokiniana* Shihira and Krauss were deposited at GenBank under the BioProject PRJNA936934. The raw sequence data were deposited in the SRA database under the accession numbers SRX19473268 (*A. protothecoides*) and SRX19473269 (*C. sorokiniana*).
